# Integrating life course perspectives into research involving people living with perinatally acquired HIV

**DOI:** 10.1186/s44263-026-00252-3

**Published:** 2026-02-11

**Authors:** Gabriela Toledo, Marthe Le Prevost, Ali Judd, Intira Jeannie Collins

**Affiliations:** 1 MRC Clinical Trials Unit, Institute of Clinical Trials & Methodology, University College London, London, UK; 2 Penta Foundation, Padua, Italy

With improved HIV treatment, growing numbers of people living with perinatally acquired HIV are reaching adulthood, yet evidence on their long-term outcomes remains limited. Expanding digital health infrastructure offers an opportunity to link paediatric and adult HIV data with broader health records to guide interventions and strengthen care.

Since 1990, an estimated 11 million children have been born with perinatally acquired HIV (PHIV). With expanding access to ART, more are surviving into adulthood. In 2023, there were an estimated 660,000 young adults aged 20–24 years living with PHIV, while the number aged over 24 years is unknown [1]. In high-income countries (HICs) with early access to antiretroviral therapy (ART) from the mid-1990s, people living with PHIV (PPHIV) are now entering their fourth and fifth decades of life, though small cohort sizes and fragmented data systems have limited the ability to fully assess life course health outcomes as this population ages. Such data from HICs and low- and middle-income countries (LMICs) are critical to shaping future paediatric and adult HIV care and informing interventions to optimise health outcomes for PPHIV globally.

While long-term survival represents a major success for PPHIV, the increased life expectancy brings new challenges. These challenges partly reflect the impact of lifelong exposure to HIV, and increasingly to ART, during critical periods of postnatal growth and development. This experience is unique to PPHIV, as behaviourally acquired HIV (BHIV) is typically acquired following physical, neurological and immunological maturity. Health and psychosocial issues at a young age may make the transition to adult services difficult, and these complexities continue to evolve beyond this stage.

Life course epidemiology provides a useful framework for understanding the long-term health trajectories of adults living with PHIV. Rather than focusing on discrete life stages, a hallmark of traditional epidemiology, a life course approach examines health across the lifespan [2]. It emphasises how the timing, duration, and accumulation of biological, clinical, and social exposures from early life onward shape outcomes in later adulthood. This perspective builds on Barker’s hypothesis of foetal programming, which proposed that foetal nutrition contributes to the risk of adult chronic diseases, including diabetes or hypertension [2]. Given the distinctive exposures associated with PHIV, applying a life course lens may enable a more accurate characterisation of emerging health issues in adulthood, and underscores the importance of large population-based longitudinal studies capable of capturing diverse data across decades of life. While research has increasingly focused on ageing with HIV [3], it is often based on adult data, with limited integration of paediatric histories for adults with PHIV.

In the United Kingdom, linkage between national paediatric HIV surveillance data and adult HIV cohort data revealed that one in ten PPHIV experienced a new AIDS diagnosis or died within five years of transitioning from paediatric to adult HIV care, with the highest risk amongst those with low CD4 counts or prior AIDS diagnoses in childhood [4]. These findings underscore the value of linking HIV data across the lifespan, demonstrating how childhood events shape adult outcomes. However, achieving such linkage requires integrated data systems. In many settings, evidence gaps persist because HIV databases remain siloed: paediatric and adult care data are often stored separately or combined without reliably capturing mode of HIV acquisition, leading to misclassification and aggregation with other populations. This fragmentation hinders consistent follow-up of PPHIV and limits the application of a life course perspective, which is key for accurately characterising ageing and comorbidity risk.

Building on this life course lens, studies of long-term wellbeing require research to move beyond the traditional HIV care cascade to capture the cumulative and interconnected nature of a broader set of outcomes across life stages. These span health (e.g. physical health markers, clinical co-morbidities, sexual and reproductive health), psychosocial (e.g. neurocognitive, mental health, quality of life) as well as socioeconomic factors (e.g. educational attainment, employment, housing stability) to assess the wider impact of HIV on health span. While some progress has been made in understanding broader markers of health as this population ages, many studies are cross-sectional, based on small sample sizes, and lack longitudinal data on clinically meaningful outcomes, as well as appropriate comparator groups. In addition, fragmented health data systems reduce opportunities to examine broader outcomes because governance and interoperability restrictions limit data linkage. This lack of integration obscures the life course picture of PHIV, and risks deepening inequities for PPHIV. Large longitudinal studies are essential to assess how risks evolve with age, to identify high-risk subgroups and timepoints, and to explore the extent to which early life events shape adult health and wellbeing.

Integrating routine health care data, such as national HIV surveillance, electronic medical records (EMRs), administrative datasets, and disease-specific registries, offers powerful opportunities to construct cohort studies and enable longitudinal analyses at a fraction of the cost of a traditional cohort study. Ageing PHIV cohorts in HICs benefit from strong surveillance infrastructures but have limited size and complex ART histories. In contrast, LMICs have larger, more contemporary PHIV cohorts alongside emerging electronic data systems. Across all settings, data governance and privacy safeguards, including legal protections, limited access protocols, and in some settings requirements for explicit consent, remain central. While these practices reflect both historical concerns about HIV-related stigma and the growing emphasis on data privacy, they can also hinder valuable public health research. As HIV care evolves and routine data become more widely used, ensuring appropriate safeguards for the ethical use of unconsented routine care data is essential to informing the needs of the ageing PPHIV population. Strong and meaningful patient and public involvement with the HIV community and wider public is crucial for this. A UK study showed that most adults living with HIV support the use of routine care data for research, provided it is done securely and with close community involvement to set research priorities and minimise stigma [5]. This reinforces the value of involving the HIV community in research, particularly in guiding ethical and acceptable uses of routine care data.

Promising efforts to bridge data siloes do exist, though are mainly focused on adults with BHIV. For instance, the Comparative Outcomes and Service Utilisation Trends (COAST) study in Canada links an HIV registry of adults aged 19 and older with a wide range of administrative health databases, including physician billing, hospital visits, cancer registries, prescriptions, and mental health data. Findings from COAST have shown a shift in cause of death from HIV-related to non-HIV-related conditions, and a higher prevalence and earlier onset of eight age-related conditions compared to a matched HIV-negative comparison group [6]. In the UK, primary care electronic records have been used to identify adults aged 18 years or older diagnosed with HIV and matched to comparable adults without HIV. Findings showed those living with HIV had a significantly higher risk of mental illness compared to people without HIV [7].

In Western Cape, South Africa, there have been similar efforts using a fully integrated data system that spans patient-level electronic records from administrative, laboratory, pharmacy, and disease management systems, resulting in rich life course data including people with and without HIV. These integrated routine data have been used to answer a range of clinical research questions, including the identification of pregnancies in youth living with PHIV, an area with sparse evidence. Compared to those with BHIV, pregnant people with PHIV pregnancies were younger, more likely to have elevated viral loads, and poorer immunological status at pregnancy onset [8], highlighting the potential benefit of enhanced monitoring of this group. Integrated data have also enabled tracking of COVID-19-related mortality among adults living with HIV, showing that HIV disease severity and unsuppressed viral loads were associated with mortality and that risk factors shifted across pandemic waves [9]. While mainly limited to higher and middle-income countries, these studies demonstrate the value of integrated health data systems for research and their crucial role in generating meaningful health insights. In high-burden, lower-resource settings, the availability and scope of large EMR datasets is increasing, driven by large investments in digitising health globally, including Malawi’s national HIV data lake and Nigeria’s Data Ecosystem [10]. Looking ahead, the shift toward integrating HIV care with wider chronic disease management will establish shared data environments and unified frameworks, offering an important opportunity to strengthen life course research on PHIV.

In summary, there is a growing population of young adults living with PHIV, whose long-term HIV and broader outcomes remain poorly understood. Addressing this gap requires embedding life course epidemiological principles into study design, incorporating patient and public involvement across the research lifecycle, and strengthening integrated digital health data systems with strong data governance frameworks to ensure ethical research. High- and middle-income countries with mature PHIV cohorts and strong data systems are well positioned to lead the development and evaluation of these frameworks, drawing on decades of follow-up across paediatric and adult care. Lessons learned from these settings can inform life course research in resource-limited, high-burden environments, strengthening global research capacity and generating evidence to improve long-term care and outcomes for PPHIV worldwide.

## Data Availability

No datasets were generated or analysed during the current study.
